# Genetic Determinants of Methicillin Resistance and Virulence among *Staphylococcus aureus* Isolates Recovered from Clinical and Surveillance Cultures in a Brazilian Teaching Hospital

**DOI:** 10.5402/2012/975143

**Published:** 2012-05-17

**Authors:** Marcus Vinicius Pimenta Rodrigues, Carlos Magno Castelo Branco Fortaleza, Camila Sena Martins Souza, Natalia Bibiana Teixeira, Maria de Lourdes Ribeiro de Souza da Cunha

**Affiliations:** ^1^Departamento de Doenças Tropicais e Diagnóstico por Imagem, Faculdade de Medicina de Botucatu, Universidade Estadual Paulista (UNESP), 18618-970 Botucatu, SP, Brazil; ^2^Departamento de Microbiologia e Imunologia, Instituto de Biociências de Botucatu, Universidade Estadual Paulista (UNESP), 18618-970 Botucatu, SP, Brazil

## Abstract

*Aims*. To quantify the presence of SCC*mec* types and virulence genes among *Staphylococcus aureus* colonizing and infecting patients from a teaching hospital. *Methods*. We analyzed 225 and 84 *S. aureus* isolates recovered from surveillance and clinical cultures, respectively. Strains were studied for the presence and type of SCC*mec*, as well as for several virulence genes. Univariate and multivariable analysis were performed in order to identify predictors of invasiveness (defined as isolation from clinical cultures). *Results*. The presence of SCC*mec* types III (OR, 2.19, 95% CI, 1.08–4.45) and IV (OR, 5.28 95% CI, 1.35–20.63) and of genes coding for exfoliative toxin B (*etb*, OR, 6.38, 95% CI, 1.48–27.46) and Panton-Valentine leukocidin (*pvl*, OR, 2.38, 95% CI, 1.16–4.86) was independently associated with invasiveness. *Conclusions*. SCC*mec* types III and IV and virulence genes are associated with greater invasiveness of *S. aureus*. Patients colonized with methicillin-resistant *S. aureus*, as well as with strains harboring *etb* or *pvl*, may be prone to develop invasive disease. Infection-preventing strategies should be more intensively applied to this group.

## 1. Introduction

 A comprehensive approach to *Staphylococcus aureus* epidemiology within healthcare settings should include the identification of reservoirs, transmission dynamics, and invasiveness. The latter issue is of major interest. While many patients are asymptomatically colonized with methicillin-susceptible (MSSA) or resistant (MRSA) *S. aureus*, only a few develop infection [1]. However, previous studies have shown that colonization is a major risk factor—or even a preceding stage—for infection [2].

 We attempted to assess determinants of infection by comparing the proportion of isolates recovered from surveillance and clinical cultures that harbored genes coding for virulence or methicillin resistance. The rational of our study was that isolates from clinical cultures, which are collected upon the diagnosis of an infectious syndrome, may be representative of “invasive” strains.

## 2. Material and Methods

The study was performed with isolates of *S. aureus *collected from October 2006 to March 2009 from hospitalized patients in the Hospital Estadual Bauru (HEB), one of the teaching hospitals from Botucatu School of Medicine. The hospital has 285 active beds, distributed among four intensive care units (ICUs), one unit for burn patients and several medical, surgical, and pediatric wards. Surveillance cultures (nasopharyngeal swabs) were routinely obtained from all patients upon admission. In ICUs and burn units, those cultures were also performed weekly thereafter. For burn patients, besides nasopharyngeal swabs, specimens were obtained from burn wound and other body sites. Clinical cultures were collected upon medical indication.

We studied isolates provided by HEB's microbiology laboratory. Whenever one patient had more than one surveillance or clinical culture positive for *S. aureus*, only the first was included in the analysis. However, if that patient presented positivity in both surveillance and clinical cultures, both isolates were analyzed. Several PCR-based methods were applied to identify virulence genes (Table 1) [3–6]. Methicillin resistance was assessed by molecular identification and typing of SCC*mec* [7]. Isolates were also submitted to strain typing through pulsed-field gel electrophoresis (PFGE) [8]. Band patterns were digitalized and analyzed with Bionumerics (Applied Maths, Belgium). Clones were defined on the basis of a similarity (Dice coefficient) greater than 0.8.

 The proportion of isolates harboring specific genes was compared through univariate statistical tests: chi-square or Fisher's exact test. Later, all results were simultaneously introduced in a single-step multivariable model (logistic regression). A *P* value of 0.05 was set as significance limit.

## 3. Results

 A total of 309 isolates were included in the study, 225 (72.8%) from surveillance cultures and 84 (27.2%) from clinical specimens. The most frequent sites of surveillance cultures were nasopharynx (59.6%), burn wound (22.7%), and oropharynx (10.2%). Among clinical specimens, blood (46.4%), wound secretions (33.3%), and tissue fragments (14.3%) predominated.

 PFGE patterns revealed a polyclonal picture. Colonizing isolates growth from surveillance cultures was grouped in 48 clones. The most disseminated clone comprised ten isolates. Forty one clones were identified among invasive isolates (i.e., those recovered from clinical cultures). The major clone grouped eight isolates. It is worth noting that in both cases, the major clones were detected all through the study period.

 Other results are summarized in Table 2. Briefly, we found an independent relation between presence of SCC*mec* types III and IV and invasiveness. Also, invasive strains were more likely to harbor genes for exfoliative toxin b (*etb*) and the Panton-Valentine leukocidin (*pvl*).

## 4. Discussion

 The finding of greater invasiveness among MRSA is not surprising. This may be partly due to direct and indirect (“populational”) ecological pressure of antimicrobial use [9]. Also, other clinical factors, such as greater severity of illness and length of stay in the hospital are both risk factors for healthcare-acquired infection and for MRSA acquisition.

 On the other hand, *pvl* lysis of leukocytes may act as a mechanism for evasion from immune response, facilitating *S. aureus* survival and tissue invasion [10]. Of note, we found *pvl *genes in 33 out of 203 SCC*mec *III and in 3 out of 9 SCC*mec *IV*-*harboring isolates. This difference did not reach statistical significance. None of the MSSA strains was positive for *pvl*.

 The finding of a relation between *etb* and invasiveness was rather puzzling. This gene was equally distributed among MSSA (3.1%) and MRSA (4.1%) isolates. It was found in 3 out of 40 isolates from blood cultures and in 4 out of 28 isolates from wound secretions. We can only hypothesize that superantigenic action may improve invasiveness. Of note, *etb *surpasses *eta *in pyrogenic activity [11].

 Of course, our results could be improved by confronting molecular results with clinical and epidemiological data. Though it was not the main objective of our study, we revised all MRSA cases in order to identify community-acquired strains. We found out that 15 (out of 220) MRSA-positive patients had positive cultures upon admission and did not refer any previous contact with healthcare settings in the last year. Among the isolates, 14 harboured SCC*mec *III and only one SCC*mec *IV. This is a starting point for further research. Still, our results point out to a role of both virulence factors and methicillin resistance in the transition from colonization to infection. The early identification of those genes among colonized patients may delimitate a group of patients deserving more intensive application of infection control measures.

## Figures and Tables

**Table 1:** List of virulence factors assessed in the study, alongside with the genes tested and the methods references.

Virulence factors	Genes	Reference
	*sea*	Johnson et al. [3]
Enterotoxins	*seb*	
	*sec-1*	
Staphylococcal toxic shock syndrome toxin 1	*tst*	

Panton-Valentine leukocidin	*LukPV*	Lina et al. [4]

Biofilm production	*icaA*	Arciola et al. [5]
	*icaD*	

Exfoliative toxins	*eta*	Johnson et al. [3]
	*etb*	
	*etd*	

Hemolysins	*hla*	Jarraud et al. [6]
	*hld*	

**Table 2:** Results of univariate and multivariable analysis of molecular predictors for invasiveness among *S. aureus* strains.

Genes	Univariate				Multivariable	
	Clinical cultures (84)	Surveillance cultures (225)	OR (95% CI)	*P*	OR (95% CI)	*P*
SCC*mec *						
Absence (reference)	14 (16.7)	75 (33.3)	Reference	—	Reference	—
type II	4 (4.8)	1 (0.4)	**21.43 (2.22–206.26)**	**0.004**	10.79 (0.95–122.14)	0.06
type III	59 (70.2)	44 (19.6)	**2.19 (1.15–4.18)**	**0.02**	**2.19 (1.08–4.45)**	**0.03**
type IV	7 (8.3)	5 (2.2)	**7.50 (2.08–27.02)**	**0.003**	**5.28 (1.35–20.63)**	**0.02**
*Virulence genes*						
*tst *	19 (22.6)	28 (12.4)	**2.06 (1.08–3.93)**	**0.03**	1.36 (0.65–2.84)	0.41
*sea *	20 (23.8)	60 (26.7)	0.86 (0.38–1.54)	0.61	1.16 (0.57–2.33)	0.69
*seb *	22 (26.2)	43 (19.1)	1.50 (0.83–2.71)	0.17	1.64 (0.85–3.16)	0.14
*sec1 *	20 (23.8)	75 (33.3)	0.63 (0.34–1.11)	0.11	0.55 (0.27–1.10)	0.09
*eta *	2 (2.4)	1 (0.4)	5.46 (0.49–61.06)	0.18	3.57 (1.48–27.46)	0.32
*etb *	8 (9.5)	4 (1.8)	**5.82 (1.70–19.86)**	**0.002**	**6.38 (1.48–27.46)**	**0.01**
*etd *	2 (2.4)	0 (0.0)	Undefined	0.07	Undefined*	0.99
*pvl *	21 (25.0)	25 (11.1)	**2.67 (1.40–5.09)**	**0.002**	**2.38 (1.16–4.86)**	**0.02**
*ica A *	80 (95.2)	220 (97.8)	0.46 (0.12–1.74)	0.26	0.71 (0.14–3.71)	0.69
*ica D *	84 (100.0)	216 (96.0)	Undefined	0.12	Undefined*	0.99
*hla *	84 (100.0)	223 (99.1)	Undefined	1.00	Undefined*	0.99
*hld *	84 (100.0)	225 (100.0)	Undefined	1.00	Not included**	not included

Note: cases are in number (%). Significant results are presented in boldface.

All variables were dichotomic except SCC*mec *(which was analyzed as a dummy variable, with absence as reference category).

OR: odds ratio. CI: confidence interval.

*CI ranging from zero to infinite. **Not included in the model, due to presence in all analyzed isolates.
